# Conserved Opposite Functions in Plant Resistance to Biotrophic and Necrotrophic Pathogens of the Immune Regulator SRFR1

**DOI:** 10.3390/ijms22126427

**Published:** 2021-06-15

**Authors:** Geon Hui Son, Jiyun Moon, Rahul Mahadev Shelake, Uyen Thi Vuong, Robert A. Ingle, Walter Gassmann, Jae-Yean Kim, Sang Hee Kim

**Affiliations:** 1 Division of Applied Life Science (BK21 Four Program), Plant Molecular Biology and Biotechnology Research Center, Gyeongsang National University, Jinju 52828, Korea; geonhees@gnu.ac.kr (G.H.S.); moon_dal@gnu.ac.kr (J.M.); rahultnau@gmail.com (R.M.S.); uyenvuong@gnu.ac.kr (U.T.V.); kimjy@gnu.ac.kr (J.-Y.K.); 2 Department of Molecular and Cell Biology, University of Cape Town, Cape Town 7700, South Africa; robert.ingle@uct.ac.za; 3 Division of Plant Sciences, Christopher S. Bond Life Sciences Center and Interdisciplinary Plant Group, University of Missouri, Columbia, MO 65211, USA; gassmannw@missouri.edu; 4 Division of Life Science, Gyeongsang National University, Jinju 52828, Korea

**Keywords:** SRFR1, CRISPR/Cas9, tomato, fungal necrotrophs, plant resistance

## Abstract

Plant immunity is mediated in large part by specific interactions between a host resistance protein and a pathogen effector protein, named effector-triggered immunity (ETI). ETI needs to be tightly controlled both positively and negatively to enable normal plant growth because constitutively activated defense responses are detrimental to the host. In previous work, we reported that mutations in *SUPPRESSOR OF rps4-RLD1* (*SRFR1*), identified in a suppressor screen, reactivated EDS1-dependent ETI to *Pseudomonas syringae* pv. *tomato* (*Pto*) DC3000. Besides, mutations in *SRFR1* boosted defense responses to the generalist chewing insect *Spodoptera exigua* and the sugar beet cyst nematode *Heterodera schachtii*. Here, we show that mutations in *SRFR1* enhance susceptibility to the fungal necrotrophs *Fusarium oxysporum* f. sp. *lycopersici (FOL)* and *Botrytis cinerea* in Arabidopsis. To translate knowledge obtained in *AtSRFR1* research to crops, we generated *SlSRFR1* alleles in tomato using a CRISPR/Cas9 system. Interestingly, *slsrfr1* mutants increased expression of SA-pathway defense genes and enhanced resistance to *Pto* DC3000. In contrast, *slsrfr1* mutants elevated susceptibility to *FOL*. Together, these data suggest that SRFR1 is functionally conserved in both Arabidopsis and tomato and functions antagonistically as a negative regulator to (hemi-) biotrophic pathogens and a positive regulator to necrotrophic pathogens.

## 1. Introduction

Plants are exposed to a wide variety of potential pathogens and developed a plethora of strategies aimed at protection. The plant immune response consists of two layers. The first layer is pattern-triggered immunity (PTI), which is induced by microbe- or pathogen-associated molecular patterns (MAMPs or PAMPs), the conserved structural molecules associated with microbial organisms, such as chitin, flagellin, and EF-Tu [1]. Recognition of MAMPs/PAMPs by pattern recognition receptors (PRRs) transduces signals to trigger downstream immune responses, such as activation of MAPK cascades, regulation of transcription factors, and expression of defense-related genes, leading to the limitation of pathogen spread and colonization [1,2]. To counteract this, pathogen effectors are injected into host cells, where they target components of the host immune system to suppress immunity [2]. Plants, in turn, evolved a second layer of defense using resistance proteins (R) to monitor pathogen effectors, called effector-triggered immunity (ETI). Many plant R proteins contain nucleotide-binding (NB) and leucine-rich repeat (LRR) domains, called NLR. NLR can be grouped into two families based on their N-terminal domains, the coiled-coil (CC)-NB-LRR (CNL) family and the Toll-interleukin 1-like receptor (TIR)-NB-LRR (TNL) family [3]. If not properly regulated, immune responses have the potential to be deleterious to the host.

Arabidopsis *SUPPRESSOR OF rps4-RLD 1* (*SRFR1*) was identified from a suppressor screen using wild-type RLD, an accession that possesses missense mutations in *RPS4* [4]. In Arabidopsis, mutations in *SRFR1* enhanced resistance to *Pseudomonas syringae* pv. *tomato* (*Pto*) DC3000 expressing *avrRps4* or *hopA1* when the corresponding *R* genes, *RPS4* and *RPS6*, are mutated, respectively [5,6]. *srfr1* mutants were equally susceptible as wild-type RLD to virulent *Pto* DC3000. The mutant *srfr1* alleles were recessive, suggesting that, genetically, *SRFR1* functions as a negative regulator of ETI [7].

SRFR1 functions as an adaptor protein by forming protein complexes containing the defense regulator ENHANCED DISEASE SUSCEPTIBILITY1 (EDS1) and resistance proteins of the TNL class, such as RPS4, RPS6, and SNC1 [8,9]. SRFR1 contains a tetratricopeptide repeat (TPR) domain that has sequence similarity to that of *Saccharomyces cerevisiae* Ssn6, which functions as a transcriptional repressor [4]. Transcript levels of defense-related genes are induced in *srfr1* mutants [5]. In addition, SRFR1 physically interacts with the immune cochaperone, suppressor of G2 allele of skp1 b (SGT1b) [8], and members of TEOSINTE BRANCHED1, CYCLOIDEA, and PCF (TCP) transcription factor family [9]. These suggest that SRFR1 functions as an adaptor protein that negatively regulates ETI-associated transcriptional immune responses in Arabidopsis.

Additionally, Arabidopsis *srfr1* (*atsrfr1*) mutants showed enhanced resistance to the generalist chewing insect *Spodoptera exigua* and the sugar beet cyst nematode *Heterodera schachtii* [10]. This, together with increased ETI to *P. syringae*, raised the possibility that SRFR1 has a broader role in determining a set-point of plant innate immunity to a wide variety of biotic stresses. Overall, characterization of SRFR1 in crops may provide potential clues to improve protection against invading plant pathogens. In this regard, advanced techniques such as genome editing offer ways to precisely edit *SRFR1* and further characterization in both model plant and crop species.

Recently, clustered regularly interspaced short palindromic repeats (CRISPR) /CRISPR-associated protein (Cas)-mediated genome editing is widely applied to genome modifications in plants [11,12]. CRISPR/Cas system includes an endonuclease Cas protein and hybrid RNA termed as single guide RNA (sgRNA). The most popular Cas protein is *Streptococcus pyogenes* Cas9 (SpCas9) that forms a complex with sgRNA and recognizes a protospacer adjacent motif (PAM) NGG in the target region [11]. The resulting protein–RNA–DNA interactions of SpCas9-sgRNA-target DNA produce double-strand breaks (DSB). Thereafter, error-prone DSB repair generates indel mutations in the target region through non-homologous end joining (NHEJ) or homology-directed repair (HDR). The application of CRISPR/Cas-based genome editing tools gained momentum to improve crop traits against plant pathogens.

Since *SRFR1* is a well-conserved single-copy gene in several crop plants, a functional study of *Solanum lycopersicum SRFR1* (*SlSRFR1*) can shed light on its role in the crop immune system. Since *atsrfr1* mutants display broad-spectrum resistance to (hemi-) biotrophic pathogens and a pest, we hypothesized that disruption of *SlSRFR1* in tomato would alter defense responses to biotic stresses. In the present work, we generated *SlSRFR1* alleles using the CRISPR/Cas9 system with two single guide RNAs (sgRNAs) that target 5′-end sequences of *SlSRFR1*. Our data reveal that mutations in *SlSRFR1* increased the expression of *PR* genes involved in salicylic acid (SA)-dependent defense signaling and suppressed the growth of the virulent bacterium *Pto* DC3000. Interestingly, mutations in *SRFR1* in both Arabidopsis and tomato enhanced susceptibility to the necrotrophic fungal pathogens *Fusarium oxysporum* f. sp. *lycopersici* (*FOL*) and *Botrytis cinerea* (*B. cinerea*). This study provides molecular insights into *SlSRFR1* function and paves the way to modulate this well-conserved gene to fine-tune plant immune responses in plants.

## 2. Results

### 2.1. Enhanced Susceptibility to the Necrotrophic Fungal Pathogens Fusarium oxysporum f. sp. lycopersici and Botrytis cinerea in Arabidopsis srfr1 Mutants

**Figure 1:**
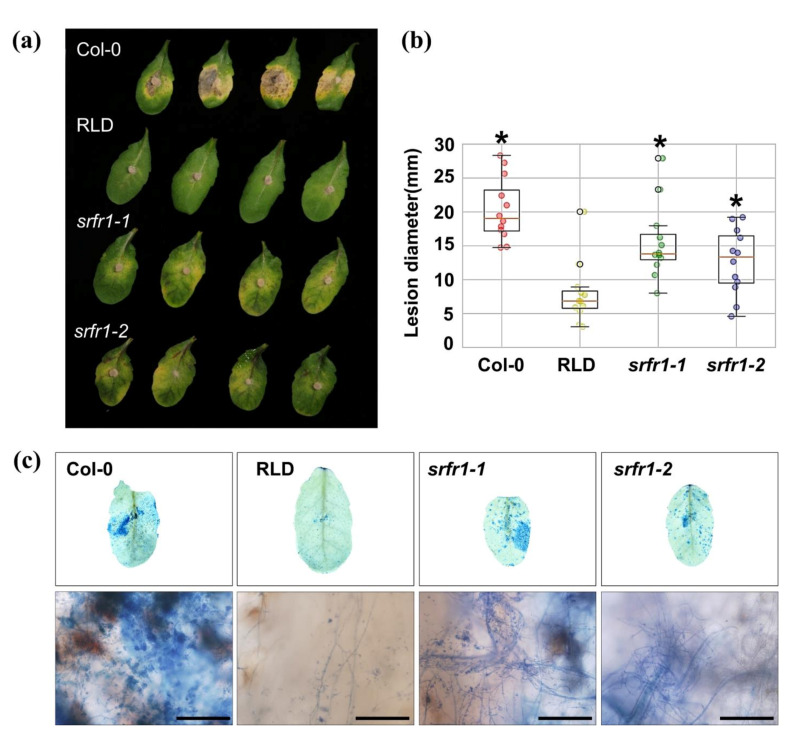
Response of Arabidopsis *srfr1* mutants to the necrotrophic fungal pathogen, *Fusarium oxysporum.* (**a**) Infection with conidia and mycelial plugs on Arabidopsis RLD, *srfr1-1,* and *srfr1-2* mutants. Detached leaves from 4-week-old plants were inoculated with 4 mm-diameter plugs of *F. oxysproum*. Photographs were taken at 14 DPI; (**b**) box plots of lesion size at 14 DPI in Col-0, RLD, *srfr1-1*, and *srfr1-2.* The y-axis displays the measured diameter of disease lesion (mm, *n* = 12) in each plant. The box ranges were determined from the twenty-fifth to the seventy-fifth percentiles. A statistically significant difference was determined by the Student’s *t*-test (* *p <* 0.05); (**c**) Leaves were collected 14 DPI with *F. oxysporum* and stained with lacto-phenol trypan blue to visualize the extent of hyphal development. The upper panel represents destained leaves with 99% ethanol, and the lower panel indicates developed fungal hyphae in each plant. Scale bar: 200 μm. This experiment was repeated once with similar results.

Arabidopsis *srfr1* mutants were mainly involved in EDS1-dependent ETI responses against *Pto* DC3000 [4,6,13] and in the resistance to chewing insect *S. exigua* and the sugar beet cyst nematode *H. schachtii*, which is an obligate biotrophic pathogen [10]. In the current study, we expanded our understanding of SRFR1-mediated plant immune responses to necrotrophic fungal pathogens. *FOL* was reported to cause vascular wilt disease in Arabidopsis and tomato. In *FOL*–plant interactions, initial fungal infection occurs primarily in the roots, resulting in disruption of vascular tissues, chlorosis, and necrosis, which leads to plant death [14,15]. To identify the role of SRFR1 in *FOL* susceptibility, the Arabidopsis wild-type accessions Col-0 and RLD and *srfr1-1* and *srfr1-2*, two recessive alleles of *SRFR1* in the RLD background, were plug-inoculated with *FOL*. The wild-type RLD was more resistant to *FOL* than Col-0 (Figure 1a). Lesion size in Col-0 was three times larger than in RLD (Figure 1b), suggesting RLD is naturally resistant to *FOL*. Interestingly, the *srfr1-1* and the *srfr1-2* mutants displayed enhanced susceptibility to *FOL* compared to RLD, as reflected by more severe symptoms, such as increased chlorosis (Figure 1a), increased lesion size (Figure 1b), and intense hyphal development (Figure 1c).

**Figure 2:**
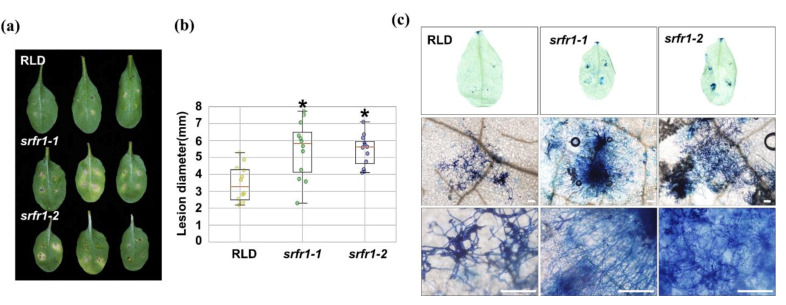
Response of Arabidopsis *srfr1* mutants to the necrotrophic fungal pathogen, *Botrytis cinerea.* (**a**) Fungal spores of *B. cinerea* grown on PDA were harvested and inoculated onto detached leaves of Arabidopsis RLD, *srfr1-1,* and *srfr1-2* mutants at a concentration of 1 × 10^8^ spores/mL. Photographs were taken at 5 DPI; (**b**) box plots of lesion size at 5 DPI in RLD, *srfr1-1*, and *srfr1-2.* The y-axis displays the measured diameter of disease lesions (mm, *n* = 12) in each plant. The box ranges were determined from the twenty-fifth to the seventy-fifth percentiles. A statistically significant difference was determined by the Student’s *t*-test (* *p <* 0.01); (**c**) leaves were collected 3 DPI with *B. cinerea* and stained with lacto-phenol trypan blue to visualize the extent of hyphal development. Scale bar: 200 μm.

Next, we asked whether mutations in *SRFR1* alter disease resistance to *B. cinerea*, which is a necrotrophic fungal pathogen that destroys plant cells at the early stage of infection, resulting in widespread tissue injury [16,17]. To answer this, the wild-type RLD and the *srfr1* mutants were drop-inoculated with *B. cinerea*. As shown in Figure 2a, the lesion areas were more extensive in *srfr1* mutants than those in RLD. The lesion area in *srfr1* mutants was twice larger than that in the wild-type (Figure 2b). Consistent with the visible symptom, *srfr1* mutants revealed more fungal hyphae aggregated near the leaf vessels compared to RLD in trypan blue staining (Figure 2c). Because accessions of Arabidopsis were reported to display isolate-specific variation of susceptibility to *B. cinerea* [18], we tested plants with a second isolate. The increased susceptibility of *srfr1-1* versus RLD to *B. cinerea* was also observed following infection with a South African pepper isolate of this necrotrophic pathogen (Figure S1). Together, these results demonstrate that mutations in *SRFR1* enhanced susceptibility to *FOL* and *B. cinerea*, indicating SRFR1 functions as a positive regulator of plant disease resistance against necrotrophic fungal pathogens.

### 2.2. Target Selection and Plasmid Vector Construction for SlSRFR1 Editing

*SRFR1* is a single-copy gene in Arabidopsis and is conserved between animals and plants [4,8]. Arabidopsis SRFR1 (AtSRFR1) consists of 11 TPR domains, which are mainly involved in protein–protein interactions [9,19]. Using the blast algorithm to annotated tomato proteome, we found that Solyc02g092780 (SlSRFR1) is the closest homolog of AtSRFR1 with 65% identity (Figure S2). Sequence alignment of AtSRFR1 and SlSRFR1 revealed that *SlSRFR1* encodes a putative protein of 1055 amino acids, and two TPR domains are positioned in the N-terminus, whereas there are nine in the central part of SlSRFR1 (Figure S2). The region of low sequence similarity between second and third TPRs (residues 133–282) is predicted to be disordered (Figure S2) [20].

We hypothesized that SRFR1 function is highly conserved between Arabidopsis and tomato based on the noticeable conservation of amino acid sequences. As a first step in determining the function of SlSRFR1, we generated *srfr1* mutants in tomato by using the CRISPR/Cas9 system. For sgRNA designing [21], standard criteria were considered, such as GC content (30–80%) and optimal secondary structure (Figure S3). SRFR1-sgRNA1 was designed to edit a site (Target 1) located towards the 5′-end of the *SlSRFR1* open reading frame. Another sgRNA, SRFR1-sgRNA2, was intended to generate indel mutations in the first TPR domain-encoding region (Target 2). Efficient editing at both targets would generate knockout alleles with deletion of a larger fragment [22].

**Figure 3:**
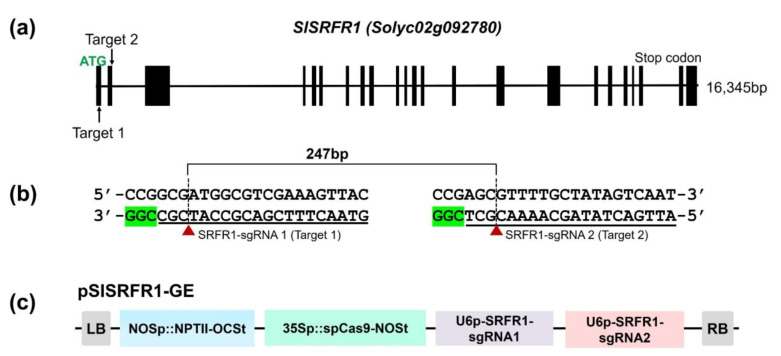
Tomato *SRFR1* gene structure and vector construction for CRISPR/Cas9-mediated genome editing in *SlSRFR1*. (**a**) Predicted *SlSRFR1* gene structure and the position of guide RNAs in *SlSRFR1*. The black box denotes exon, and the line means intron. Two guide RNAs are indicated with an arrow in the first and the second exons, respectively; (**b**) target sequences of sgRNAs. Predicted cut sites by CRISPR/Cas9 are denoted with red triangles and dotted lines; (**c**) construction of CRISPR/Cas9-mediated genome editing for *SRFR1* in tomato. NPTII was used for a plant selection marker. Two guide RNAs were expressed by AtU6 promoter and terminated by poly T.

Golden Gate cloning strategy [23] was used to assemble four independent expression modules into a single plant binary T-DNA vector (*pSlSRFR1-GE*) that included a plant selection marker (*neomycin phosphotransferase* (*nptII*), kanamycin resistance), functional SpCas9 expression cassette, and two sgRNA expression cassettes (Figure 3c). Finally, *pSlSRFR1-GE* was transformed into tomato cultivar M82 using Agrobacterium-mediated transformation to produce genome-edited plants by optimized tissue culture conditions [24,25].

### 2.3. Generation and Analysis of SlSRFR1 Alleles Created by CRISPR/Cas9

**Figure 4:**
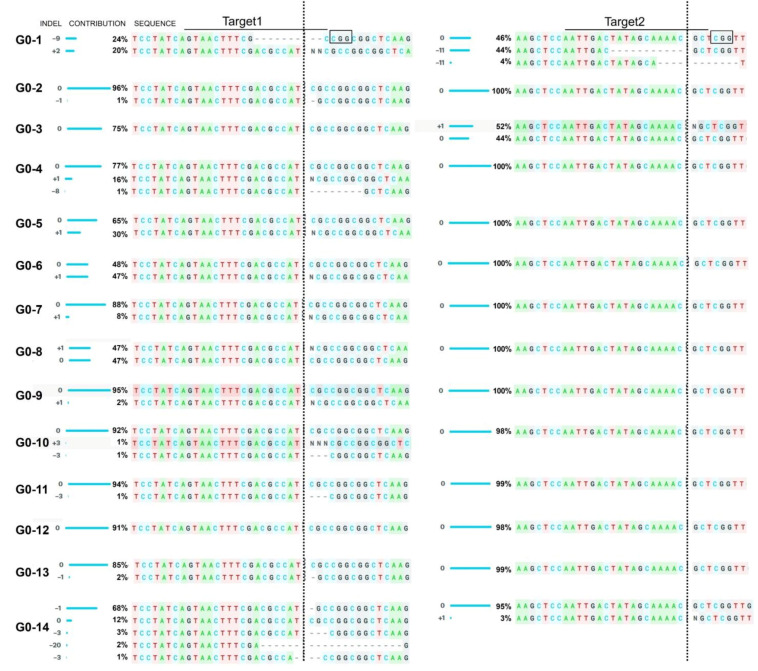
ICE analysis with amplified PCR fragments from *slsrfr1* G0 plants. PCR fragments were amplified using genomic DNA isolated from *slsrfr1* G0 plants, and the sequences were analyzed by ICE analysis with Sanger sequencing results. The ICE analysis represents the indel pattern in each target sequence of guide RNA, the contribution of each edited pattern, and the genome-edited sequences. Black box indicates the PAM site and the target sequences of guide RNAs are overlined. The ICE contribution values may not add up to 100% based on Sanger sequencing data of a specific sample, because the remaining percent of the decomposed data does not fit in predicted outcomes by ICE program. Vertical stippled lines represent cleavage site by SpCas9 for each sgRNA target. The analyzed sequences are shown in 3’-to-5’ direction through ICE analysis with Sanger sequencing results.

In the first generation (G0), 37 plants were regenerated from transformed cotyledons. Genomic DNA was isolated from the generated plants to verify potential genome editing events. The targeted DNA region was amplified using gene-specific primers by polymerase chain reaction (PCR). PCR products amplified from wild-type M82 and G0 leaves showed similar size after electrophoretic separation on the agarose gel, indicating no larger deletion occurred in the targeted area. Sanger sequencing data derived from PCR amplicons were subjected to decomposition analysis using Inference of CRISPR Edits (ICE) online tool [26]. Among the analyzed plants, indel mutations were detected in 14 G0 plants, resulting in a CRISPR/Cas9-mediated editing efficiency of 37.84% (Figure 4).

All indel mutations occurred at the cleavage point (3 bp upstream of the PAM site) of SpCas9 in target sites of both sgRNAs. The insertion of 1 bp (G0-5, G0-6, G0-7, G0-8, and G0-9) and the deletion of 3 bp (G0-10 and G0-11) were mainly observed in the SRFR1-sgRNA1-targeted site located immediately upstream of the start codon (Figure 4). Five independent events (G0-5 to 9) showed similar editing patterns, i.e., insertion of 1 bp at the cutting site of SpCas9. The presence of mixed peaks in ICE-based decomposition analysis of PCR amplicons confirmed the presence of heterozygous *slsrfr1* mutants in G0 generation. Therefore, three G0 lines (G0-1, G0-2, and G0-3) were further characterized in the next generation (G1) to obtain homozygous plants. PCR amplicons were analyzed by cleaved amplified polymorphic sequences (CAPS) assay (Figure 3 and Figure S4). Since the mutations were introduced within *Bcg*I (Target 1) and *Bcc*I (Target 2) recognition sites, restriction digestion patterns distinguished homozygous, heterozygous, and wild-type plants. Full-length PCR fragments containing Target 2 (945 bp) and Target 1 (453 bp) were digested by *Bcg*I and *Bcc*I enzyme, respectively. By the polymorphism, the amplified templates were not digested by *Bcg*I and *Bcc*I in homozygotes G1 plants.

**Figure 5:**
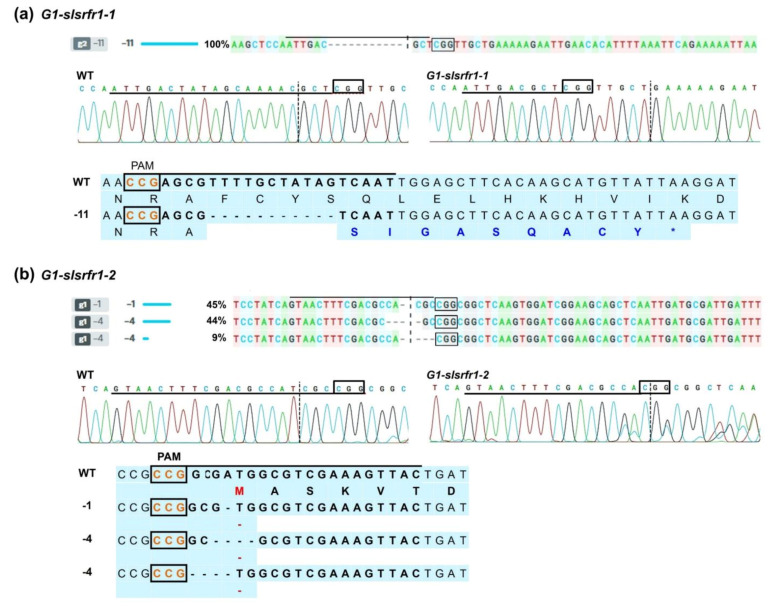
ICE analysis with amplified PCR fragments from *slsrfr1* G1 plants, Sanger sequencing chromatograms in target sequence, and predicted amino acid sequences in two homozygous *G1-slsrfr1* plants. (**a**) Eleven nucleotide deletions in *slsrfr1-1* produces a premature stop codon, colored with blue; (**b**) 1 or 4 nucleotide deletions in *slsrfr1-2* remove a start codon (M), colored with red. Nucleotide deletions are indicated by dashes (-). The PAM sites are boxed, and the target sequences of guide RNAs are overlined.

Additionally, all homozygous G1 plants from CAPS assay were examined by deep DNA or Sanger sequencing (Figure 5, Figure S5, and Table S1). Successful genome editing produced four types of G1 plants possessing different alleles, including two monoallelic homozygotes (11 bp deletion, *slsrfr1-1*, and one bp insertion in TPR domain, *slsrfr1-3*) and two biallelic homozygotes (1 and 4 bp deletion, *slsrfr1-2*, and 22 bp deletion and 4 bp deletion removing the start codon, *slsrfr1-4*) (Figure 5, Figure S5, and Table S1). Insertion of 1 bp or 11 bp deletion in TPR domain generated premature stop codon (Figure 5a and Figure S5a). Additionally, the mutation in *slsrfr1-2* and *slsrfr1-4* was identified in the start codon (Figure 5b and Figure S5b). Although off-targeting is not a significant concern in plant studies [27], potential off-targets predicted for both the sgRNAs were analyzed in the G1 line. Target loci containing off-target sites were amplified by PCR. Sanger sequencing analysis of purified PCR amplicons showed no detectable editing in any of the examined off-target sites (Table S2).

### 2.4. Altered Morphology and Expression of Defense Marker Genes in CRISPR/Cas9-Edited slsrfr1 Plants

**Figure 6:**
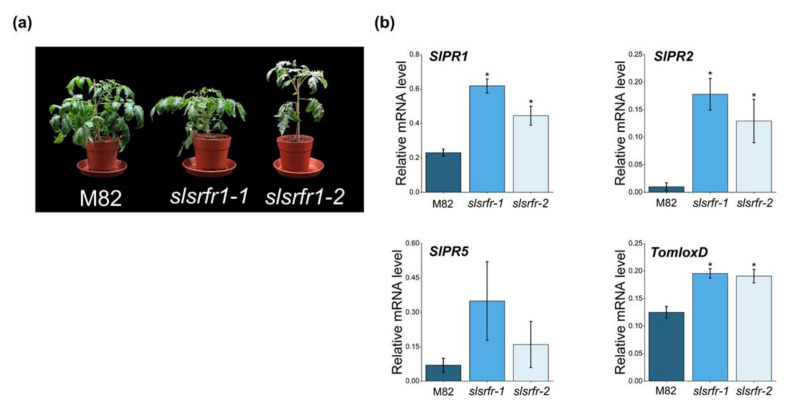
Altered morphology and expression of defense marker genes in CRISPR/Cas9-edited *slsrfr1* plants. (**a**) Growth phenotype of 6-week-old M82 and *slsrfr1* lines grown in 16 h light/8 h dark long-day photoperiod; (**b**) relative mRNA expression of defense-related genes. The genes used for gene expression analysis refer to *SlPR1 (Solyc09g007010.1), SlPR2 (Solyc01g008620.2), SlPR5(Solyc08g080640),* and *TomloxD (Solyc03g122340.2).* Gene expression levels of each gene were normalized with *SlACT (Solyc04g011500.3.1)* as an internal control. Error bars represent standard deviation. A statistically significant difference was determined by the Student’s *t*-test (* p < 0.01).

In Arabidopsis, RLD *srfr1-1* and *srfr1-2* mutants exhibit normal and wild-type-like morphology, although there is a slight decrease in growth. However, as shown in *srfr1-4*, a mutation in *SRFR1* in Col-0 leads to extreme stunting and abnormal growth because of the constitutive activation of the Col-0-specific resistance gene *SNC1* [28]. *G1-slsrfr1* plants showed weak growth reduction, not severe stunting, reminiscent of RLD *srfr1* phenotype (Figure 6a). *G1-slsrfr1* morphology leads us to test whether the expression of defense marker genes is altered in *slsrfr1* mutants. As shown in Figure 6b, as with Arabidopsis *srfr1* mutants [5], *SlPR1* and *SlPR2* expressions were significantly increased in two independent *G1-slsrfr1* mutants, *slsrfr1-1* and *slsrfr1-2*, compared to wild-type M82. Consistent with this, SlPR1 protein was strongly accumulated in *G1-slsrfr1-1* lines (Figure S6). These suggest that CRISPR/Cas9-mediated mutations in *SlSRFR1* upregulate the expression level of SA-dependent defense markers both transcriptionally and translationally. In addition, consistent with *atsrfr1,* a JA biosynthetic gene (*TomloxD*) and JA-responsive genes *(TD and SlPR4* genes) were induced in *slsrfr1* mutants (Figure 6b and Figure S8) [10], suggesting both SA- and JA-dependent defenses are upregulated in untreated CRISPR/Cas9-edited *slsrfr1* lines.

### 2.5. Enhanced Resistance to Pto DC3000 in CRISPR/Cas9-Edited slsrfr1 Plants

**Figure 7:**
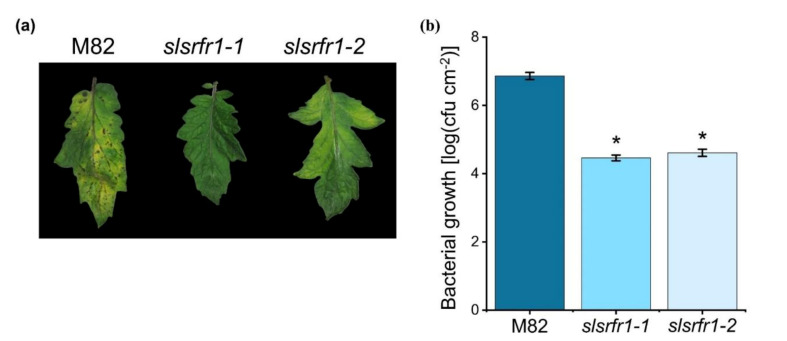
Response of *slsrfr1* lines to the bacterial pathogen *Pseudomonas syringae* pv. *tomato* DC3000. (**a**) Disease symptoms of parental M82 (left), *slsrfr1-1* (middle), and *slsrfr1-2* (right) dip-inoculated with *Pto* DC3000. Disease symptoms (leaf chlorosis) were recorded at 5 DPI. Only the fourth trifoliate leaflet of the plants was inoculated; (**b**) in planta bacterial growth was measured in indicated plant lines on day 5 after inoculation with *Pto* DC3000 at a density of 2 × 10^8^ cfu/mL. Values represent averages of cfu/cm^2^ leaf tissue from four replicas, and error bars denote standard deviation. Asterisks indicate that the growth of DC3000 was significantly different between M82 and *slsrfr1* mutants as determined by a two-tailed Student’s *t*-test (* *p* < 0.01). This experiment was repeated twice with similar results.

The absence of increased resistance to virulent *Pto* DC3000 is observed in Arabidopsis *srfr1* mutants, even though defense-related genes are constitutively upregulated [5,28]. To test whether the level of resistance to virulent bacteria is altered in *slsrfr1* mutants, we challenged 6-week-old *G1- slsrfr1* and wild-type M82 plants with *Pto* DC3000 by partial dipping method. As shown in Figure 7, both *slsrfr1-1* and *slsrfr1-2* leaves showed enhanced resistance to *Pto* DC3000 in contrast to wild-type M82. Disease symptoms in M82, such as chlorosis and water-soaked lesions, were dramatically reduced in *G1- slsrfr1* lines. Consistent with the visible symptoms, *slsrfr1-1* and *slsrfr1-2* showed approximately 100-fold lower *Pto* DC3000 growth than wild-type M82 (Figure 7b). These results suggest that mutations in *SlSRFR1* increased SA-pathway defense genes, leading to enhanced resistance against the (hemi-)biotrophic pathogen *Pto* DC3000.

### 2.6. Enhanced Susceptibility to Fusarium oxysporum f. sp. lycopersici in CRISPR/Cas9-Edited slsrfr1 Plants

**Figure 8:**
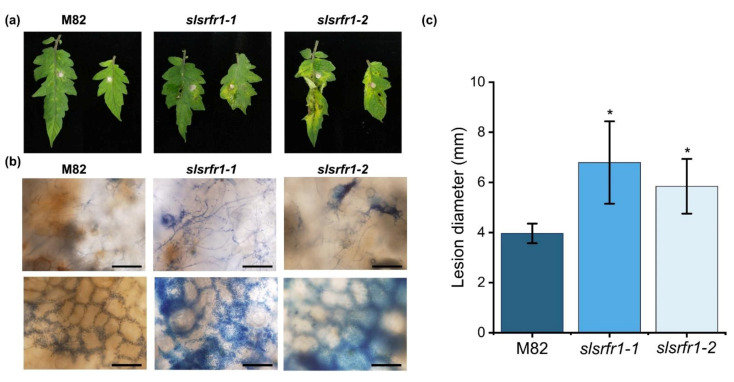
Plant response to *Fusarium oxysporum* f. sp. *lycopersici* in *slsrfr1* lines. Plant response was analyzed at 3 days after plug inoculation of *FOL* in *slsrfr1* lines. (**a**) Detached leaves from 6-week-old M82, *slsrfr1-1,* and *slsrfr1-2* were inoculated with 4 mm-diameter plugs of *F. oxysproum*. Photographs were taken at **3** DPI; (**b**) Trypan blue staining in *FOL*-inoculated *slsrfr1* lines. The bar represents 200 μm in the upper layer and 100 μm in the lower layer; (**c**) lesion size at 3 DPI in *slsrfr1* lines. Plant response of *slsrfr1-1* and *slsrfr1-2* against *FOL* was repeated three times and once, respectively, with similar results. A statistically significant difference was determined by the Student’s *t*-test (* *p < 0.01*).

As shown in Figure 1, the Arabidopsis RLD *srfr1* mutants display enhanced susceptibility against *FOL* and *B. cinerea*. To test the functional conservation of SRFR1 in tomato, we analyzed the resistance response of *G1-slsrfr1* lines after inoculation with the necrotrophic fungal pathogen *FOL*. Three days after plug-inoculation with *FOL*, all *slsrfr1* mutants, *slsrfr1-1, slsrfr1-2, slsrfr1-3,* and *slsrfr1-4*, displayed enhanced susceptibility compared to wild-type M82, as indicated by severe necrosis and enlarged lesion area (Figure 8a,c, and Figure S7b). Trypan blue staining of the infected leaves showed that extensive development of fungal hyphae was observed in *slsrfr1-1* and *slsrfr1-2* with expanding lesion areas (Figure 8b). These results demonstrate that SlSRFR1 positively regulates the immune response against the necrotrophic pathogen *FOL,* and that SRFR1 function is conserved between Arabidopsis and tomato.

## 3. Discussion

Gene gain-of-function or loss-of-function mutants are important resources for functional studies in plant biology. Previous studies documented that Arabidopsis RLD *srfr1* mutants showed enhanced resistance to *Pto* DC3000 expressing *avrRps4* and *hopA1*, the generalist chewing insect *S. exigua* and the sugar beet cyst nematode *H. schachtii* [4,6,10,28]. *SRFR1* is well-conserved in different organisms [4] and exists as a single-copy gene in many crop plants, including tomato. Tomato is a major horticultural crop plant, and tissue culture-based genetic engineering protocols are well adopted for precise genome manipulations [24,25]. Despite intensive research in host–microbe interactions, molecular interactions are not yet entirely known in crop plants. Therefore, the study of proteins/pathways implicated in plant–microbe crosstalk, especially in crop–pathogen interaction, would facilitate basic understanding and crop improvement [29].

In the current work, CRISPR/Cas9-mediated genome editing was successfully applied to produce *slsrfr1* mutant alleles to understand its function. SRFR1 forms an immune complex with TNL type R proteins and TCP TFs in Arabidopsis [8,13,28]. The first TPR domain is present at the beginning of the N-terminus in both Arabidopsis and tomato SRFR1. Accordingly, we targeted two sites, including the start codon and the first TPR domain-encoding region of the *SlSRFR1* gene, for CRISPR-mediated mutant generation. Typically, indel mutations were introduced between the third and the fourth bp of sgRNA counting from the PAM site. Indel generation patterns at targeted sites indicated a higher editing rate at Target 1 (SRFR1-sgRNA1) than Target 2 (SRFR1-sgRNA2). A difference in editing activity resulted from a combination of factors reported recently in *SlPelo1*, for example, unstable Cas9-sgRNA complex formation, target accessibility, and Cas9 availability while using multiple sgRNAs [25]. Although the genome editing efficiency was lower in the G0 stage generating biallelic and chimeric editing patterns, screening of segregating populations produced homozygous *slsrfr1* mutant lines in G1 generation. Independent *G1-slsrfr1* lines harboring early stop codon (*slsrfr1-1* and *slsrfr1-3*) and no-start-codon (*slsrfr1-2* and *slsrfr1-4*) were successfully generated. Although Col-0 *srfr1-4* exhibited an autoimmune-like stunting phenotype by activation of Col-0 specific *SNC1*, RLD *srfr1* did not show growth defect; nonetheless, the shoot weight was slightly reduced [6]. As with Arabidopsis RLD *srfr1*, the growth of *slsrfr1* was slightly diminished compared with wild-type M82, reflecting tomato may not possess a functional Arabidopsis *SNC1* ortholog. However, we could not exclude the possibility that a *SNC1* ortholog is weakly expressed in tomato. It would be interesting to identify whether functional *SNC1* is conserved in tomato.

Plant resistance responses against a biotrophic pathogen are generally mediated by induction of the SA-signaling pathway and are partly due to the hypersensitive response. On the other hand, resistance to necrotrophic pathogens is dependent on the JA and/or the ethylene signaling pathway, suggesting the opposing mechanism of plant immune responses to two different pathogens with distinct attack strategies [30]. To explore the role of SRFR1 in responses to necrotrophic fungal pathogens, we performed pathogenesis assays in wild-type RLD, *atsrfr1-1,* and *atsrfr1-2* by inoculating with *FOL* and *B. cinerea*. The *atsrfr1* mutants showed enhanced susceptibility with increased necrosis, enhanced development of fungal hyphae, and expanded lesion areas compared to wild-type in response to invading necrotrophic fungal pathogens. The transcripts of TNL type *R* genes and *PR* genes were upregulated in the *atsrfr1* mutants [4,5], suggesting boosted SA-pathway defense genes in *atsrfr1* might suppress the plant defense against a necrotrophic fungal pathogen. In addition, it was shown previously that *atsrfr1-1* mutants display a downregulation of the JA/ethylene response pathway genes *ORA59* and *PDF1.2* in response to the chewing insect *S. exigua*, whereas, at resting state, these genes were upregulated in *atsrfr1-1*. At the same time, *atsrfr1-1* showed upregulation of the JA pathway gene *VSP2* upon induction by *S. exigua*. This correlated with increased resistance of *atsrfr1-1* to insect herbivory. The increased mRNA levels of JA/ethylene genes in uninduced *slsrfr1* mutants and the increased susceptibility of Arabidopsis and tomato *srfr1* mutants to necrotrophic pathogens reported here are consistent with these earlier studies and highlight the conservation of *SRFR1* functions on multiple levels.

Besides, in Arabidopsis, there are several well-studied mutants that show enhanced SA defense signaling and suppressed JA defense signaling [31]. For instance, a mutation in *BOTRYTIS-INDUCED KINASE1* (*BIK1*) exhibited elevated SA accumulation and SA-pathway defense genes and attenuated expression of *PDF1.2a*, leading to increased resistance to *Pto* DC3000 and enhanced susceptibility to *B. cinerea* [32]. Loss-of-function in *BOTRYTIS SUSCEPTIBLE 1 (BOS1)* resulted in growth inhibition of *Pto* DC3000 but increased susceptibility to the necrotrophic pathogens *B. cinerea* and *Alternaria brassicicola* [33]. Furthermore, a *wrky33* mutant displayed boosted susceptibility to *B. cinerea* compared to wild-type with increased levels of SA-pathway genes and SA accumulation [34]. In tomato, pre-treatment of SA enhanced the development of a necrotrophic pathogen, *Alternaria solani* [35]. Moreover, Hernández-Aparicio and colleagues showed that a susceptible tomato cultivar displayed elevated SA levels in untreated plants and increased expression of *PR1* and of the ethylene signaling gene *1-aminocyclopropane-1-carboxylate synthase* 2 (*ACS2*) in response to *FOL* compared with a resistant tomato cultivar [36]. Consistent with these, our findings indicate that the expression level of *SlPR1,* *SlPR2,* and *SlPR5* in the SA response pathway was upregulated, and resistance response to *Pto* DC3000 was enhanced in the CRISPR/Cas9-induced *slsrfr1* mutants. Conversely, susceptibility of *slsrfr1* and *atsrfr1* mutants was promoted in response to necrotrophic fungal pathogens. The antagonistic role of SRFR1 to pathogens with different attack strategies allows us to set up a new model for SRFR1; it functions as a negative regulator to (hemi-) biotrophic pathogens and a positive regulator to necrotrophic pathogens in plant disease resistance, and its function is possibly conserved in most crop plants.

Indeed, our data indicate that the function of SRFR1 in tomato and Arabidopsis is evolutionarily conserved. Interestingly, Arabidopsis SRFR1 was reported to associate physically with TCP transcription factors and interact with the TOPLESS family genetically, which likely allows SRFR1 to function as a negative regulator in plant immunity [9,37]. These findings combined with our functional analysis of tomato SRFR1 suggest that SlSRFR1 potentially interacts with some transcription factors acting as positive regulators of tomato resistance against (hemi-) biotrophic pathogens to sequester them away from defense gene promoters. To further validate the role of SlSRFR1 in SA-based defense, determining the concentrations of endogenous SA in *slsrfr1* mutants is of particular interest. On the other hand, based on the positive function of SRFR1 in the JA signaling pathway, we suggest that SRFR1 may coordinate with JA-responsive proteins. It would be fascinating to identify whether SRFR1 interacts with JA biosynthesis enzymes and transcriptional regulators inducing JA-responsive genes. To further investigate the role of SRFR1 in JA-based defense, measurements of the expression of JA-responsive genes and endogenous JA concentration are required.

In this study, we focused on the characterization of *SlSRFR1* using CRISPR-mediated *slsrfr1* mutants with two different targets in the genome. One (*slsrfr1-1* and *slsrfr1-3*) produced an early stop codon early in the open reading frame, and the other (*slsrfr1-2* and *slsrfr1-4*) removed the start codon of *SRFR1*. In the future, the development of additional *SlSRFR1* alleles with indel mutations in the 5′-UTR of *SlSRFR1*, which is designed to produce weak alleles, will help to establish the functional aspects in tomato. Because of the antagonistic function, SRFR1 appears as a novel example of a switch model. In detail, plants need to decide to activate or inhibit the role of SRFR1 in response to different pathogens. In the future, there will be powerful technologies to conditionally suppress or enhance SRFR1 function in the plant to protect crops from pathogens. For the ultimate goal of engineering durable and broad-spectrum resistance in crop plants, it is therefore necessary to improve our knowledge of the fundamental principles of plant immune system regulation.

## 4. Materials and Methods

### 4.1. Construction to Generate Tomato SRFR1 Alleles

CRISPR-P v2.0 program generated available target sequences of SpCas9-guide RNA complex in N-terminal *SlSRFR1* [38]. Among the candidates in the CRISPR-P tool, the two sgRNAs were selected based on the parameters such as on-score, optimal secondary structure, GC contents, and possible off-target sequences. Two guide RNAs with Arabidopsis *U6* promoter (originated from Addgene #46968) and poly T were generated by primer dimerization, and they were inserted into Level 1 plasmids *pICH47751* and *pICH47761*, respectively. A plant selection marker (Addgene #51144), *SpCas9* (Addgene #49771), guide RNAs driven by the *AtU6* promoter, linker (Addgene #48019) was assembled into pAGM4723 by the Golden Gate cloning system [23].

### 4.2. Tomato Transformation Mediated by Agrobacterium and Generation of CRISPR/Cas9-Edited slsrfr1 Plants

Tomato wild-type M82 was described previously [39]. *Agrobacterium tumefaciens* GV3101 (MP90)-mediated transformation was conducted to deliver T-DNA containing a plant selection marker, SpCas9, and two guide RNA assemble into tomato. The transformation was followed as described [24,25]. Tomato cultivar M82 was grown on 1/2 MSO media at 25 °C in a 16 h light/8 h dark photoperiod for 7 days. The cotyledon fragments were prepared by cutting and incubated on PREMC media in the dark for a day. The plant binary plasmid containing CRISPR components was transformed into *A. tumefaciens* GV3101 (MP90) by electroporation. The 3 mL of 18 h grown bacterial culture was subcultured into 30 mL of LB (50 mg L^−1^ kanamycin and 10 mg L^−1^ gentamicin). The bacterial grown cells (OD600 = 0.8~1) were harvested at 3000 rpm for 15 min and suspended in 30 mL of liquid ABM-MS medium (pH 5.2) containing 200 μM acetosyringone (#D134406, Sigma, St. Louis, MO, USA). Next, tomato cotyledon fragments were added into 1 h grown culture and incubated for 20 min. The cotyledon fragments were placed on ABM-MS containing 200 μM acetosyringone and then incubated at 25 °C for 2 days in darkness. The co-cultivated explants were washed with ddH_2_O followed by with 500 mg L^−1^ timentin by gently shaking for 2 min. The remaining water on the surface of the explants was removed using the Whatman papers. The callus from transformed explants was induced on a selection media (SEL4-70) and was transferred onto SEL4-70 every two weeks until the true shoot was identified. The true shoots were moved onto root induction media (RIM) to induce roots. Healthy plants were placed on the soil and grown at 25 °C in a 16 h light/8 h dark photoperiod.

### 4.3. Isolation of Genomic DNA from Tomato

Genomic DNA extraction was followed with modification as described [40]. Two leaf discs were collected from individual G0 and G1 plants using a cork borer (No. 5) and were frozen in liquid nitrogen. The leaf discs were ground by mixer mil (#MM301, Retsch, Haan, Germany) with beads for 1 min. Ground tissues were suspended in 300 μL 2X CTAB extraction buffer (0.1 M Tris, 2% CTAB, 1.4 M NaCl, and 20 mM EDTA) and were incubated at 65 °C for 20 min. After the addition of 300 μL Chloroform into suspended tissues, they were vigorously mixed and centrifugated at 14000 rpm for 5 min. The 200 μL of supernatants were transferred into a new Eppendorf tube. The genomic DNA was precipitated with 200 μL isopropanol for 2 min at room temperature and centrifugated at 14,000 rpm for 5 min. The pellets were washed with 1 mL of 70% EtOH by inverting and then centrifugated at 14000 rpm for 1 min. The pellets were dried at room temperature for 10 min. The pellets were suspended with 50 μL of ddH_2_O. Among them, 2 μL of genomic DNA were used in 25 μL of PCR reaction volume.

### 4.4. Analysis of Editing Activities by Sequencing and Cleaved Amplified Polymorphic Sequence (CAPS) Assay

Target regions of the *SlSRFR1* locus were PCR-amplified using gene-specific primers (Table S6) and sequenced at Cosmo Genetech. ICE (Inference of CRISPR Edits), and SnapGene (GSL Biotech LLC) tools were used for Sanger sequencing data analysis. Targeted deep sequencing was conducted for an accurate genome editing analysis in G1-*slsrfr1* lines. The 945 bp PCR products were generated using SlSRFR1-F1/SlSRFR1-R2 from genomic DNA as a template. The second (155 bp and 150 bp) PCR fragments were amplified using appropriate primer sets (Table S6). Final PCR products were amplified with dual Index adapters (combination of D501~D508 and D701~D712) using second PCR products as templates. Primer sequences for third PCR were pre-determined by manufacturers to assign unique IDs. Purified DNA fragments were analyzed at the MiSeq sequencing service (MiniSeqTM System, Illumina, USA). Indel mutations in SlSRFR1 alleles were valued with the CAS-analyzer program (http://www.rgenome.net/ accessed on 2 May 2021).

For CAPS analysis, SlSRFR1 fragments were amplified with appropriate primer sets (Table S6) for SlSRFR1-sgRNA1 (Target 1) and SlSRFR1-sgRNA2 (Target 2). Thereafter, 5 μL of PCR products were incubated with *Bcc*I or *Bcg*I at 37 °C for 3–4 h. Genotyping by CAPS assay of genome-edited plants was determined by the type of DNA band pattern on agarose gel electrophoresis of digested PCR products.

### 4.5. Bacterial and Fungal Pathogenesis Assay

The disease assay with *Pseudomonas syringae* pv. *tomato (Pto)* DC3000 was conducted as previously described [41]. Briefly, *Pto* DC3000 containing the *pVSP61* empty vector was grown in Pseudomonas agar plates containing kanamycin (50 mgL^−1^) and rifamycin (50 mgL^−1^) at 28 °C. The third or the fourth leaflets of 6-week-old tomato were dipped into bacterial suspensions of 2 × 10^8^ CFU/mL for 30 s and maintained the high humidity by the plastic bag. Leaf discs with a total area of 1 cm^2^ per sample were ground in 10 mM MgCl_2_, and solutions were plated in serial dilutions on a selective media (4 replicas) at 5 days post-inoculation. Statistical comparison of bacterial growth was tested using a two-tailed Student’s *t*-test.

Plug inoculation for *Fusarium oxysporum* f. sp. *lycopersici* (*FOL*) on the detached leaves was followed as described [42]. *FOL* (saccardo) Snyder & Hansen from Korean Agricultural Culture collection 40037 was grown on potato dextrose (#254920, BD Difco, Franklin Lakes, NJ, USA) with 1.5% agar at 30 °C in the dark for 3 days, and the plate was incubated to 25 °C (16 h light/8 h dark) for 5 days. Small plugs (4 mm) were placed on detached 4-week-old Arabidopsis and 6-week-old tomato leaves. Arabidopsis leaves were incubated at 22 °C (11 h light/13 h dark) for 14 days, and tomato leaves were incubated at 25 °C (16 h light/8 h dark) for 3-8 days. Arabidopsis wild-types Col-0 and RLD and *srfr1-1* and *srfr1-2* were described previously [4].

A Korean isolate of *Botrytis cinerea* was cultured on potato dextrose agar at 22 °C in the dark for 5 days, and fungal spores were harvested with ddH_2_O and counted. Detached leaf bioassay was performed by dropping 10 μL of 1 × 10^8^ spores/mL onto 4-week-old Arabidopsis leaves. Inoculated Arabidopsis leaves were incubated at 22 °C in 11 h light/13 h dark for 3 to 5 days.

### 4.6. Trypan Blue Staining

Trypan blue staining was performed as described [43]. Arabidopsis leaf and a small piece of tomato tissue infected with fungal pathogens were immersed into Trypan blue solution (1:1:1 = lactic acid (85% *w*:*w*):phenol (pH 8.0):glycerol (≧ 99%) with a final concentration of 10 mg/mL of Trypan blue) and incubated by gently shaking for 1 h, and Trypan blue solution and chlorophyll were removed with 99% ethanol during the overnight.

### 4.7. RNA Isolation and cDNA Synthesis

Three leaf discs from *SlSRFR1* alleles with a cork borer (No. 5) were collected into 2 mL of Eppendorf tube and immediately frozen with liquid nitrogen. Total RNA was isolated from tissue powder using RiboEx (#301-001, GeneAll, Seoul, South Korea) by following the manufacturer’s instructions. Genomic DNA was removed by TURBO DNA-free kit (#AM1907, Invitrogen, Carlsbad, CA, USA). cDNA synthesis was conducted using SuperiorScript III cDNA synthesis kit (#EZ405S, Enzynomics, Daejeon, SouthKorea) according to the manufacturer’s instructions.

### 4.8. Gene Expression Analysis by qRT-PCR

The relative gene expression was quantified by qRT-PCR. The primers [44,45,46,47,48] used in this study are listed in Table S6. The expression level of the other genes of interest was normalized by *SlACT* and *SlGAPDH*. The PCR reactions were processed on the CFX384 system (BioRad, Hercules, CA, USA) using the QuantiNova SYBR® Green PCR Kit (#208054, Qiagen, Germantown, MD, USA) with the following conditions: 95 °C for 2 min and 40 cycles of 95 °C for 5 s, 60 °C for 15 s.

### 4.9. Immunoblot Analysis

Two leaf discs collected from tomato tissues with a cork borer (No. 5) were ground in 50 µL of 8 M urea buffer to extract total protein, as described previously [49]. Plant debris was centrifuged twice at 12,000 rpm for 10 min, and the collected supernatant was separated on 12% polyacrylamide gel. Immunoblot analysis was performed using α-PR1 antibody (1:10000 dilution, #AS10 687, Agrisera, Vännäs, SWEDEN) or α-Actin antibody (1:10000 dilution, #AS13 2640, Agrisera, Vännäs, SWEDEN). Primary antibody was applied to the membrane at 4 °C with shaking overnight. A secondary anti-Rabbit antibody (1:10000 dilution, #W4011, Promega, Madison, WI, USA) was treated at room temperature with shaking for 1 h and washed 4 times with 1× TBST. Tomato PR1 and ACTIN proteins were visualized by the mixture of Clarity Western ECL Substrate (#1705061, Bio-Rad, Hercules, CA, USA) and SuperSignal™ West Femto Maximum Sensitivity Substrate (#34094, Thermo Scientific™, Waltham, MA, USA).

## 5. Conclusions

Plant immune responses, especially effector-triggered immunity, must be tightly controlled to provide a delicate balance of growth and defense. In Arabidopsis, SUPPRESSOR OF rps4-RLD 1 (SRFR1) functions as a negative regulator not only in EDS1-dependent ETI to *Pseudomonas syringae* pv *tomato (Pto*) DC3000 but also in defense responses to the generalist chewing insect *Spodoptera exigua* and the sugar beet cyst nematode *Heterodera schachtii*. In addition, we showed here that SRFR1 functions as a positive regulator of plant disease resistance against the necrotrophic fungal pathogens *Fusarium oxysporum* f. sp. *lycopersici* (*FOL*) and *Botrytis cinerea*. In addition, we translated research initiated with the model plant Arabidopsis to the crop plant tomato. Tomato *slsrfr1* mutants generated using the CRISPR/Cas9 system displayed opposite disease resistance to a (hemi-) biotrophic pathogen and a necrotrophic pathogen. Mutations in *SlSRFR1* upregulated SA-pathway defense genes and enhanced resistance to *Pto* DC3000, whereas they elevated susceptibility to *FOL*. Overall, our data reveal a novel finding of the antagonistic function of SRFR1 in both Arabidopsis and tomato and functional conservation in these two plants. The CRISPR/Cas9 based-mutations in *SRFR1* in tomato will lead us to study plant responses to economically important pathogens and pests and the mechanisms of growth and defense trade-off in crops. Besides, the selection of homozygous G2 generations will allow us to develop non-GMO, non-transgenic tomato *srfr1* mutants for durable and broadspectrum resistance.

## 6. Patents

The application of patent is being planned on the basis of the results identified in this study.

## Data Availability

Not applicable.

## Supplementary Materials

The following are available online at https://www.mdpi.com/article/10.3390/ijms22126427/s1, Figure S1: The Arabidopsis *srfr1-1* mutant displays increased susceptibility to a South African pepper isolate of *Botrytis cinereal*, Figure S2: Comparison of the deduced amino acid sequence of SRFR1 from Arabidopsis (*Arabidopsis thaliana*, AT4G37460) with rice (*Oryza sativa*, LOC_Os07g02300.1), tomato (*Solanum lycopersicum*, Solyc02g092780.1), potato (*Solanum tuberosum*, Sotub02g031240.1.1), and pepper (*Capsicum annuum,* XP_016560980.1), Figure S3: Secondary structures of sgRNAs predicted using Mfold online server, Figure S4: CAPS analysis with *SlSRFR1*-*sgRNA1-* and -*sgRNA2*-derived G1 mutants, Figure S5: Sequence alignment of *G1-slsrfr1* mutants and wild-type M82, Figure S6: Constitutive accumulation of tomato PR1 in individual *G1*-*slsrfr1-1* plants, Figure S7: Plant response to *Fusarium oxysporum* f. sp. *lycopersici* in *SlSRFR1* alleles, Figure S8: Expression of JA-responsive genes in CRISPR/Cas9-edited *slsrfr1* plants, Table S1: Genome editing contribution in *slsrfr1* G0 plants, Table S2: Off-targeted data in all *SlSRFR1* alleles, Table S3: The *SRFR1* single guide RNA and target sequences used in the study, Table S4: Potential off-targets of single guide RNAs used in this study, Table S5: Primers used in the off-target analysis, Table S6: Primers used in this study.
