# Supplementary material for: Conserved Opposite Functions in Plant Resistance to Biotrophic and Necrotrophic Pathogens of the Immune Regulator SRFR1
Source: Int J Mol Sci. 2021 Jun 15;22(12):6427. doi: 10.3390/ijms22126427 (PMC8233967; doi:10.3390/ijms22126427)
Supplement: Supplementary file 1 [file ijms-22-06427-s001.zip › ijms-1233729-supplementary.pdf]

Supplementary Materials

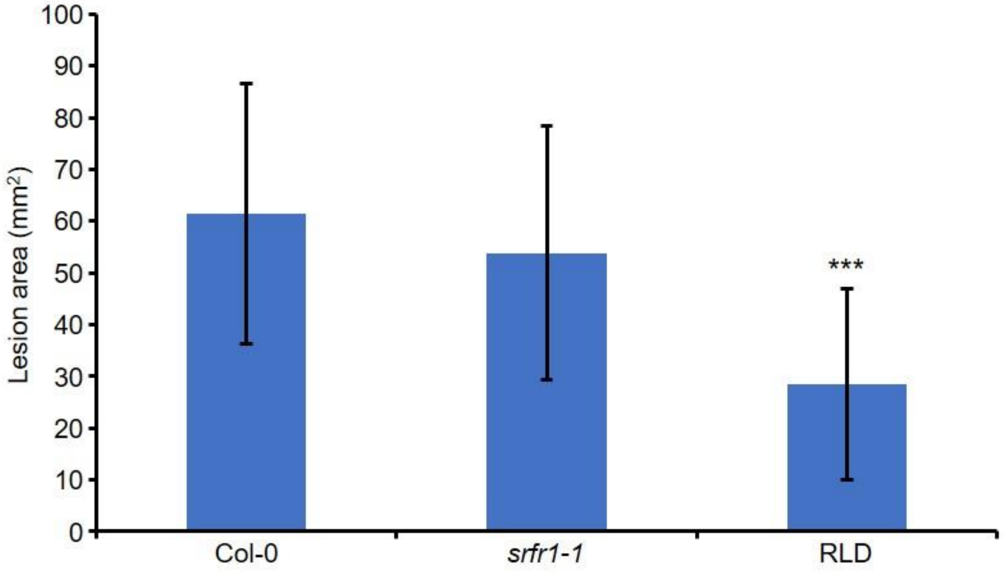

Figure S1. The Arabidopsis *srfr1-1* mutant displays increased susceptibility to a South African pepper isolate of *Botrytis cinerea*. Detached leaves from 4-week-old plants grown under a 16 h light/8 h dark cycle at 22 °C were inoculated at dawn with 10 μL of half-strength grape juice containing 5×10^4 *B. cinerea* (pepper isolate) spores mL^{-1} and lesion areas measured after 72 h using ImageJ. Data shown are mean lesion sizes ± standard deviation from 48 (Col-0), 47 (*srfr1-1*) and 41 (RLD) detached leaves. Asterisks denote a significant difference of values for RLD compared to those for Col-0 and *srfr1-1* at *** $p < 0.0001$ as determined by Tukey's HSD test.

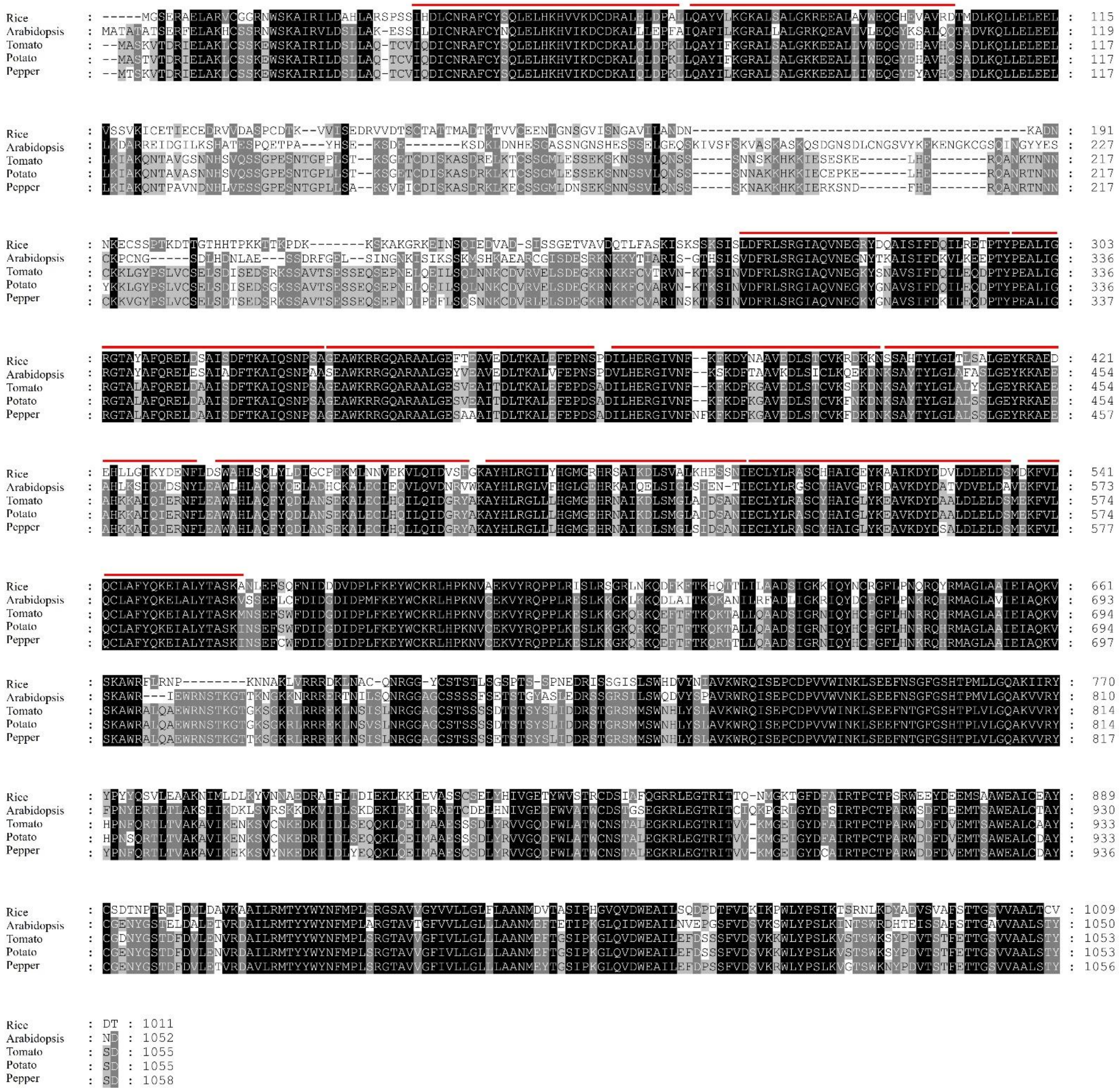

Figure S2. Comparison of the deduced amino acid sequence of SRFR1 from Arabidopsis (*Arabidopsis thaliana*, AT4G37460) with rice (*Oryza sativa*, LOC_Os07g02300.1), tomato (*Solanum lycopersicum*, Solyc02g092780.1)), potato (*Solanum tuberosum*, Sotub02g031240.1.1), and pepper (*Capsicum annuum*, XP_016560980.1). Black shading shows amino acids conserved in all entries. The gaps introduced to maximize alignment are indicated by dashes (-). Redline represents a tetratricopeptide repeat (TPR) domain. The alignment of amino acid sequences was performed with GeneDoc 2.7.

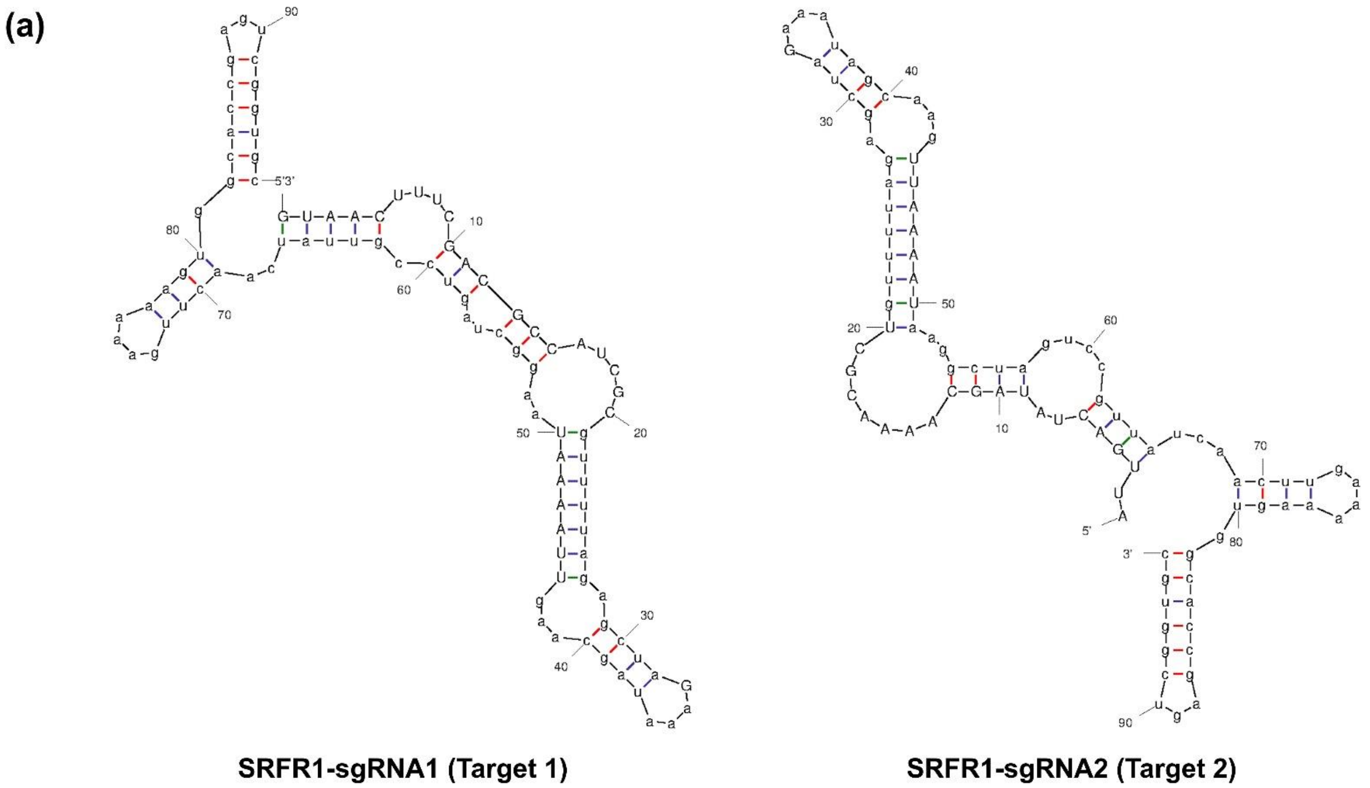

(b)

sgRNA	TSL (3)	GSL (0)	CBP (>7)	TBP (>12)	IBP (>6)	GC (30-80%)
SRFR1-sgRNA1	3	0	5	11	0	55
SRFR1-sgRNA2	3	0	4	9	0	35

Figure S3. Secondary structures of sgRNAs predicted using Mfold online server. (**a**) Target sgRNA secondary structure; (**b**) Parameters calculated from the predicted secondary structures of sgRNAs. TSL: total stem-loop; GSL: Stem-loop in the guide sequence; CBP: consecutive base pair (guide sequence and the other sequence); TBP: total base pairs in guide sequence with other sequences; IBP: internal base pairs in sgRNA; GC content (%) of sgRNA. Further details about the criteria available on the webpage of the CRISPR-P 2.0 tool (http://crispr.hzau.edu.cn/CRISPR2/).

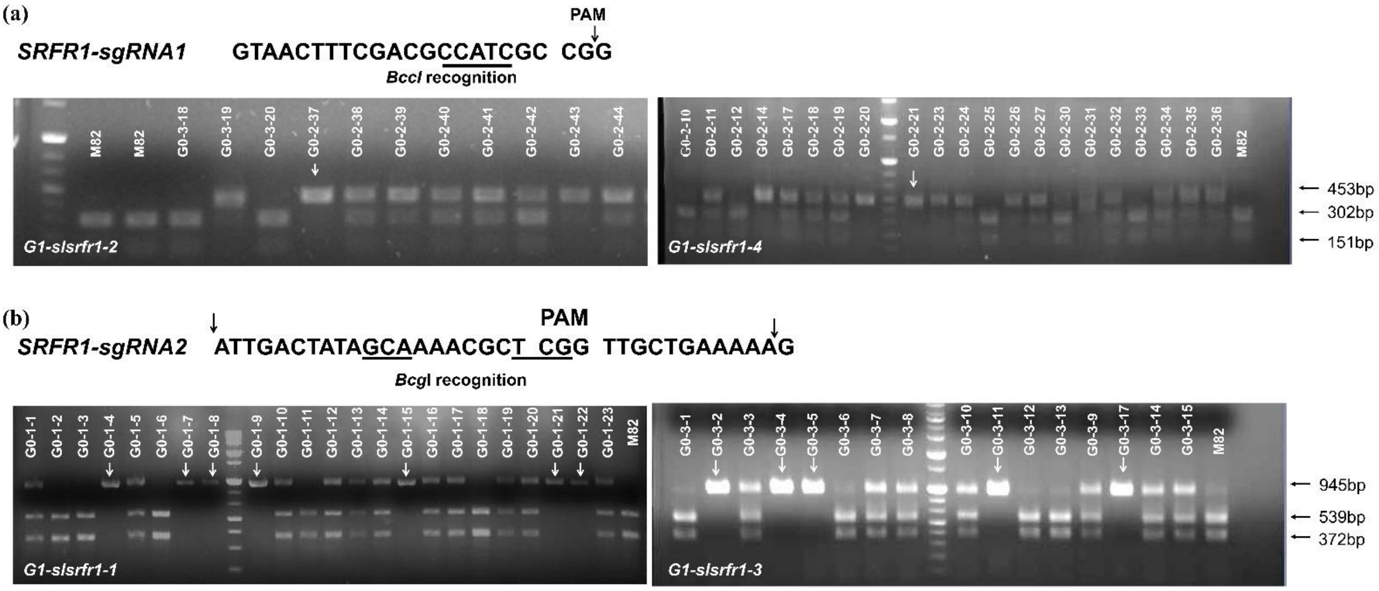

Figure S4. CAPS analysis with *SlSRFR1-sgRNA1-* and *-sgRNA2*-derived G1 mutants. Homozygote *slsrfr1-2* and *slsrfr1-4* (**a**) and *slsrfr1-1* and *slsrfr1-3* (**b**) were determined from CAPS analysis by *Bcc*I and *Bcg*I restriction enzyme, respectively. Full-length 453 bp PCR fragment was amplified for genotyping in the *SlSRFR1-sgRNA1* target area (top), and full-length 945 bp PCR fragment for selecting homozygotes in the *SlSRFR1-sgRNA2* target area (bottom). Amplified PCR fragments were digested with *Bcc*I and *Bcg*I, respectively. Black arrows represent cutting sites by indicated restriction enzymes. White arrows indicate homozygous mutants.

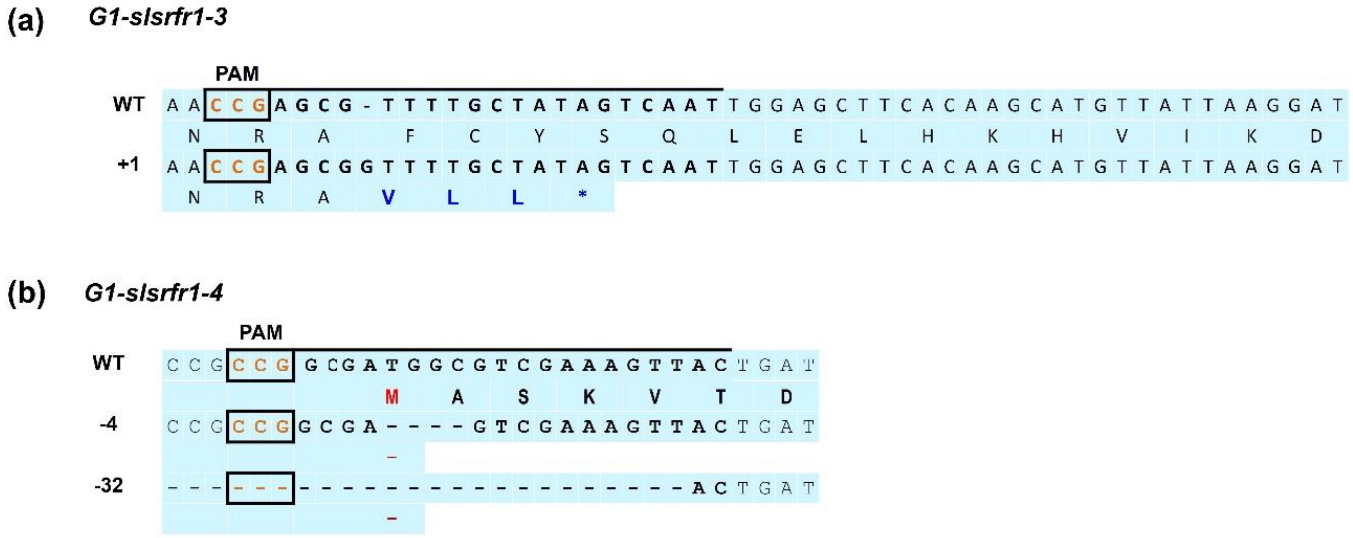

Figure S5. Sequence alignment of *G1-slsrfr1* mutants and wild-type M82. Mutations in *slsrfr1-3* and *slsrfr1-4* were determined by Illumina MiSeq sequencing. (**a**) One nucleotide insertion in *slsrfr1-3* produces a premature stop codon, colored with blue; (**b**) 4 or 32 nucleotide deletions in *slsrfr1-4* removes a start codon (M) colored with red. Nucleotide deletions are indicated by dashes (-). PAM sites are boxed-in, and target sequences of guide RNAs are overlined.

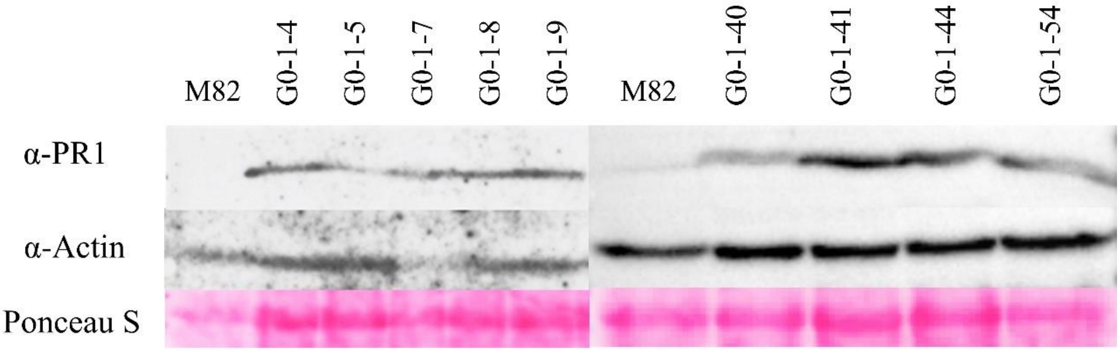

Figure S6. Constitutive accumulation of tomato PR1 in individual *G1-slsrfr1-1* plants. Proteins were extracted from 6-week-old *slsrfr1-1* G1 plants and were detected by western blot using α-PR1 and α-Actin (an internal control).

(a)

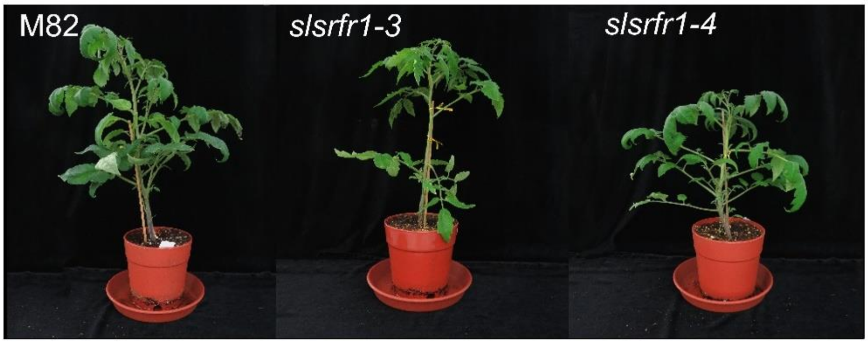

(b)

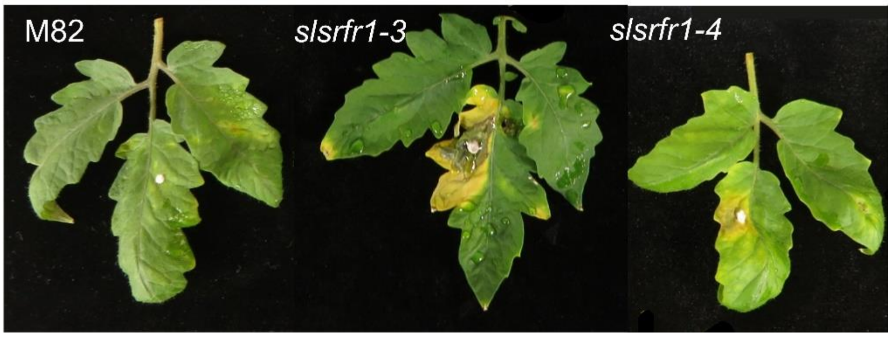

Figure S7. Plant response to *Fusarium oxysporum* f. sp. *lycopersici* in *SlSRFR1* alleles. (**a**) Morphology of 6-week-old M82, *slsrfr1-3* and *srfr1-4* grown at 25 °C in 16 h light/8 h dark long-day photo-period; (**b**) Detached leaves from 6-week-old M82, *slsrfr1-3,* and *slsrfr1-4* were inoculated with 4 mm-diameter plugs of *F. oxysproum*. Photographs were taken at 8 DPI.

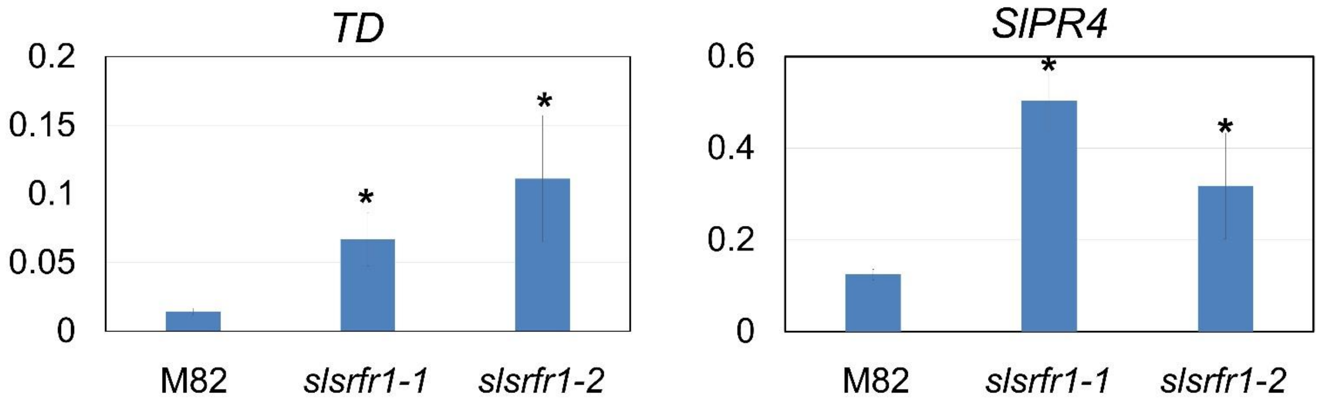

Figure S8. Expression of JA-responsive genes in CRISPR/Cas9-edited *slsrfr1* plants. The genes used for gene expression analysis refer *to TD (Solyc09g008670.2) and SlPR4 (Solyc01g097240.2)*. Gene expression levels of each gene were normalized with *SlACT (Solyc04g011500.3.1)* as an internal control. Error bars represent standard deviation. A statistically significant difference was determined by the Student's t-test (* $p < 0.05$).

Table S1. Genome editing contribution in *slsrfr1* G0 plants.

No.	G0 alleles	Indel frequency	Homozygote G1 plants identified from CAPS analysis	G1 alleles	Positon of editing	Type of event	Homozygote alleles/total analyzed lines
1	G0-1	60%	G0-1-4,7,8,9,15,21,22,40,41,44,54	*slsrfr1-1*	sgRNA2	-11 nt	11/57
2	G0-2	1%	G0-2-37	*slsrfr1-2*	sgRNA1	-1nt/-4nt	1/44
3	G0-3	44%	G0-3-2,4,5,11,17	*slsrfr1-3*	sgRNA2	+1 nt	5/20
4	G0-2	1%	G0-2-21	*slsrfr1-4*	sgRNA1	-32nt/-4nt	1/44

Table S2. Off-targeted data in all *SlSRFR1* alleles.

No.	off target candidates / Alleles	Indel (%)					
		sgRNA1-OFF_1	sgRNA1-OFF_2	sgRNA2-OFF_1	sgRNA2-OFF_2	sgRNA2-OFF_3	sgRNA2-OFF_4
1	*slsrfr1-1*	0	0	0	0	0	0
2	*slsrfr1-2*	0	0	0	0	0	0
3	*slsrfr1-3*	0	0	Failed	0	0	0
4	*slsrfr1-4*	0	0	0	0	0	0

Table S3. The *SRFR1* single guide RNA and target sequences used in the study.

**No.**	**Name**	**gRNAs**	**Target position on genome**	**Number of off-targets (<4 MMs)**
1	sgRNA1	GTAACTTTCGACGCCATCGC	5′UTR and CDS	1
2	sgRNA2	ATTGACTATAGCAAAACGCT	CDS	3

Table S4. Potential off-targets of single guide RNAs used in this study.

Potential off-target sequence	Number of mismatch	Locus	Gene	Region
sgRNA1 : GTAACTTTCGAAAACATCGC				
GTAACCTTCGAAGTCATCGA	4	SL3.0ch08:-31939614		Intergenic
GTAACTTTCAAGGCCATCGA	3	SL3.0ch05:+48464484		Intergenic
GAAACTTTCGAAAACATCGC	4	SL3.0ch08:+55060271		Intergenic
GTGAATATCGACGCCATCGA	4	SL3.0ch01:+65466561		Intergenic
GAAACTTTCGACCTCATAGC	4	SL3.0ch07:+66595791		Intergenic
GTAACTTTCGAAGGCTTTGC	4	SL3.0ch02:+35442157	gene:Solyc02g062950.3	CDS
sgRNA2 : ATTGACTATAGCAAAACGCT				
ATTGACTAAAACAAAAAACT	4	SL3.0ch05:+5657976		Intergenic
AATGAATATAGAAAAAAGCT	4	SL3.0ch01:-65718384		Intergenic
ATTGAATATAGCAAAATGTT	3	SL3.0ch03:+67920252		Intergenic
ATTGATTATAGTAAAACACA	4	SL3.0ch12:+66227955	gene:Solyc12g096230.2	utr
ATTTACTACAGCAAAACATT	4	SL3.0ch04:-39705061	gene:Solyc04g049130.3	intron
TTAGACTATAGCAATACACT	4	SL3.0ch09:-48437258		Intergenic
TTAGACTATAGCAATACACT	4	SL3.0ch10:-22995255		Intergenic
ATTGACTATAGCACAAAAAT	4	SL3.0ch10:+12194723		Intergenic
TTTGACTATAGAAACAAGCT	4	SL3.0ch04:+62510472		Intergenic
ATGGACTATAGCAAAAAAAT	4	SL3.0ch06:+3388631		Intergenic
ATTGAATATACCTAAACCCT	4	SL3.0ch11:+36073500		Intergenic
ATTGACAATAACAAAAGGCT	3	SL3.0ch09:-46995239		Intergenic
ATTGACTATAGAAAAAGTTT	4	SL3.0ch11:+13491883		Intergenic
ATTTAGTAAAGCAAAAGGCT	4	SL3.0ch04:+7910625		Intergenic
GTTGACTATAGCAAAACGAC	3	SL3.0ch10:+17248976		Intergenic
ATTGACTCTAGCGAAAGGAT	4	SL3.0ch07:+13077343		Intergenic
ATTGACTATTGCCAACCTCT	4	SL3.0ch12:-62098880		Intergenic
ATTGACTATTCCCAATCGCT	4	SL3.0ch07:-36501993		Intergenic
AGTGACTATACCAAATTGCT	4	SL3.0ch00:-19086437	gene:Solyc00g247300.3	CDS
AGTGACTATACCAAATTGCT	4	SL3.0ch04:+52095679	gene:Solyc04g054257.1	CDS

The mismatched sequences are indicated by red

Table S5. Primers used in the off-target analysis.

Name	Sequences (5′→3′)	Guide RNA	Potential off-target sequences
sgRNA1-OFF-1-F	GTCGTCGAATGCCTCCATCAGTC	sgRNA1 : GTAACTTTCGAAAACATCGC	GTAACCTTCGAAGTCATCGA
sgRNA1-OFF-1-R	GTTATTCACGAGTGGCGAGCAC		
sgRNA1-OFF-2-F	GTAGCTCCAGCACCATAATAAGCC		GTAACTTTCGAAGGCTTTGC
sgRNA1-OFF-2-R	CAAGAAAAAGTCCTCGCCGC		
sgRNA2-OFF_1-F	ATCGTATTCAACATTAGCCTTACTGAGAAG	sgRNA2 : ATTGACTATAGCAAAACGCT	ATTGAATATAGCAAAATGTT
sgRNA2-OFF_1-R	AATTGCACAGGGAGGGGAAAC		
sgRNA2-OFF_2-F	TGGTAAAGAGCCTCACATGATGACC		GTTGACTATAGCAAAACGAC
sgRNA2-OFF_2-R	GTCTATGGGCTTGTAAAAGACCATTG		
sgRNA2-OFF_3-F	CGGATGCCCTAGTTCAAGGTG		ATTTACTACAGCAAAACATT
sgRNA2-OFF_3-R	GGAGAGATTCAATGACACTAAGAAGCTG		
sgRNA2-OFF_4-F	TTTCCAAAATCAGTTGTGTTGTAGCC		AGTGACTATACCAAATTGCT
sgRNA2-OFF_4-R	GACGCCGATAAGTTTATGCCAGAG		

Table S6. Primers used in this study.

No.	Name	Sequence (5′-3′)	Purpose
1	SlSRFR1-F1	CGGTCAATAAAACACGTGTC	Genotyping
2	SlSRFR1-R2	GAGCTTCATCCTACTCCTCG	Genotyping
3	SlSRFR1BccI-R	GGAACAGTGAAAAACGTAAC	Genotyping
4	SlSRFR1-MiSeq-1-F2	ACACTCTTTCCCTACACGACGCTCTTCCGATCTAATCGCATCAATTGAGCTGC	MiSeq
5	SlSRFR1-MiSeq-1-R2	GTGACTGGAGTTCAGACGTGTGCTCTTCCGATCTATGACGCAAGTTTGAGCAAG	MiSeq
6	SlSRFR1-MiSeq-2-F2	ACACTCTTTCCCTACACGACGCTCTTCCGATCTTTGGACATTTATGAATGAACTGATTAA	MiSeq
7	SlSRFR1-MiSeq-2-R2	GTGACTGGAGTTCAGACGTGTGCTCTTCCGATCTTAGGATCGAGCTGAAGTGCC	MiSeq
8	crU6C9-F1	CAGTCGGTCTCAGGAGTGATCAAAAGTCCCACATCGATCAGGTGATATATAGCAGCTTAGTTTA	SgRNA construction
9	crU6C9-R1	CAGTCGGTCTCACAATCGCTATGTCGACTCTATCATTATATAAACTAAGCTGCTATATATCACC	SgRNA construction
10	9gR-SlSRFR1-g5-F1	CAGTCGGTCTCAATTGGTAACTTTCGACGCCATCGCGTTTTAGAGCTAGAAATAGCAAGTTAAAATAAGGCTAGTCCGTTATCAAC	SgRNA construction
11	9gR-R1	CAGTCGGTCTCAAGCGAAAAAAAGCACCGACTCGGTGCCACTTTTTCAAGTTGATAACGGACTAGCCTTATTTT	SgRNA construction
12	9gR-SlSRFR1-g10-F1	CAGTCGGTCTCAATTGGATTGACTATAGCAAAACGCTGTTTTAGAGCTAGAAATAGCAAGTTAAAATAAGGCTAGTCCGTTATCAAC	SgRNA construction
13	SlPR1-qRT-F	GCCATATTTCACTCTTGTGAG	qRT-PCR
14	SlPR1-qRT-R	TAGTCTGGCCTCTCGGACA	qRT-PCR
15	SlPR2-qRT-F	TCCAGGTAGAGACAGTGGTAAA	qRT-PCR
16	SlPR2-qRT-R	CTGGTTTCTTTGGTGTTCCTGC	qRT-PCR
17	SlPR5-qRT-F	GCAACAACTGTCCATACACC	qRT-PCR
18	SlPR5-qRT-R	AGACTCCACCACAATCACC	qRT-PCR
19	TomloxD-qRT-F	GACAAGCAATAGCAGGAGTG	qRT-PCR
20	TomloxD-qRT-R	TAAGTGTGCCAACATCAGAC	qRT-PCR
21	SlACT-qRT-F	GAAATAGCATAAGATGGCAGACG	qRT-PCR
22	SlACT-qRT-R	ATACCCACCATCACACCAGTAT	qRT-PCR
23	SlGAPDH-qRT-F	CTGCTCTCTCAGTAGCCAACAC	qRT-PCR
24	SlGAPDH-qRT-R	CTTCCTCCAATAGCAGAGGTTT	qRT-PCR
25	TD-qRT-F	AGCTCAAACACACGCGCTGGA	qRT-PCR
26	TD-qRT-R	AACCCCCACCACCAACAGGT	qRT-PCR
27	SlPR4-qRT-F	CGATCTAAATTGATTTCATAGTACG	qRT-PCR
28	SlPR4-qRT-R	TCGTGAAGGATATACAAAATACA	qRT-PCR
